# Impact of High Salt Diet on Cerebral Vascular Function and Stroke in *Tff3*^−/−^*/*C57BL/6N Knockout and WT (C57BL/6N) Control Mice

**DOI:** 10.3390/ijms20205188

**Published:** 2019-10-19

**Authors:** Nataša Kozina, Zrinka Mihaljević, Mirela Baus Lončar, Martina Mihalj, Mihael Mišir, Marina Dobrivojević Radmilović, Helena Justić, Srećko Gajović, Kate Šešelja, Iva Bazina, Anita Horvatić, Anita Matić, Nikola Bijelić, Edi Rođak, Ivana Jukić, Ines Drenjančević

**Affiliations:** 1 Faculty of Medicine Osijek, University Josip Juraj Strossmayer Osijek, Institute and Dept of Physiology and Immunology, J. Huttlera 4, HR-31000 Osijek, Croatia; natasa.kozina@mefos.hr (N.K.); zrinka.mihaljevic@mefos.hr (Z.M.); martina.mihalj@mefos.hr (M.M.); acosic@mefos.hr (A.M.); ivana.jukic@mefos.hr (I.J.); 2 Ruđer Bošković Institute, Department of Molecular Medicine; Bijenička 54, HR-10000 Zagreb, Croatiakseselja@irb.hr (K.Š.); ibazina@irb.hr (I.B.); 3 Clinical Hospital Center Osijek, Dept of Dermatology and Venerology, J. Huttlera 4, HR-31000 Osijek, Croatia; 4 Clinical Hospital Center Osijek, Neurology Clinic, J. Huttlera 4, HR-31000 Osijek, Croatia; mihaelmisir@gmail.com; 5 University of Zagreb, School of Medicine, Croatian Institute for Brain Research, Zagreb, Croatia, Šalata 12, HR-10000 Zagreb, Croatia; marina.radmilovic@mef.hr (M.D.R.); helena.justic@mef.hr (H.J.); srecko.gajovic@hiim.hr (S.G.); 6 Proteomics laboratory, Faculty of Veterinary Medicine, University of Zagreb, Heinzelova 55 HR-10000 Zagreb, Croatia; horvatic.ani@gmail.com; 7 Faculty of Medicine Osijek, University Josip Juraj Strossmayer Osijek, Institute and Dept of Histology and Embriology, J. Huttlera 4, HR-31000 Osijek, Croatia; nbijelic@mefos.hr (N.B.); erodak@mefos.hr (E.R.)

**Keywords:** *Tff3* gene, flow-induced dilation, high salt diet, stroke

## Abstract

High salt (HS) dietary intake leads to impaired vascular endothelium-dependent responses to various physiological stimuli, some of which are mediated by arachidonic acid (AA) metabolites. Transgenic *Tff3^−/−^* gene knockout mice (*Tff3^−/−^/*C57BL/6N) have changes in lipid metabolism which may affect vascular function and outcomes of stroke. We aimed to study the effects of one week of HS diet (4% NaCl) on vascular function and stroke induced by transient occlusion of middle cerebral artery in *Tff3^−/−^* and wild type (WT/C57BL/6N) mice. Flow-induced dilation (FID) of carotid artery was reduced in WT-HS mice, but not affected in *Tff3^−/−^-*HS mice. Nitric oxide (NO) mediated FID. NO production was decreased with HS diet. On the contrary, acetylcholine-induced dilation was significantly decreased in *Tff3^−/−^* mice on both diets and WT-HS mice. HS intake and *Tff3* gene depletion affected the structural components of the vessels. Proteomic analysis revealed a significant effect of *Tff3* gene deficiency on HS diet-induced changes in neuronal structural proteins and acute innate immune response proteins’ expression and *Tff3* depletion, but HS diet did not increase the stroke volume, which is related to proteome modification and upregulation of genes involved mainly in cellular antioxidative defense. In conclusion, *Tff3* depletion seems to partially impair vascular function and worsen the outcomes of stroke, which is moderately affected by HS diet.

## 1. Introduction

High salt (HS) dietary intake modifies the vascular reactivity to different physiological stimuli in different vascular beds [1,2,3,4], including cerebral circulation, affecting the magnitude of responses, as well as the mechanisms underlying vascular reactivity, even in normotensive animals and humans [5,6]. For example, cerebral resistance arteries of healthy male Sprague–Dawley (SD) rats on a short-term 4% NaCl (HS) diet exhibited decreased endothelium-dependent flow-induced vasodilation (FID). Furthermore, the FID mechanisms became entirely dependent on nitric oxide (NO) in the HS group, as opposed to the low-salt diet group, where FID was dependent on NO, prostanoids, and epoxyeicosatrienoic acid [7]. FID represents an important physiological regulator of tissue perfusion, including cerebral circulation [8,9,10]. Altered vasoreactivity is associated with cerebral infarction [11]. Many biological processes involving the interaction of neurons, glia, vascular cells and matrix components, increased oxidative stress, and inflammation have an important role in tissue injury and repair and are involved in the pathogenesis and consequences of stroke [12]. The changes that take place in coronary and cerebral vessels are mirrored by carotid arteries, as has been documented by experimental and clinical studies [13,14]. Thus, the carotid artery represents a good model for studying all complex processes involved in development and outcomes of stroke.

Genetically modified mice are frequently used stroke models for studying the molecular pathophysiology of stroke [15,16,17,18]. The *Tff3^−/−^/*C57BL/6N (*Tff3*) knockout mice model represents a promising new model for studying vascular function, atherosclerotic lesions, and the synthesis of proinflammatory cytokines due to altered lipid metabolism [19,20]. The metabolites of arachidonic acid (AA) have a significant role in mediating vascular reactivity to various stimuli, affecting tissue perfusion and supply, and exerting proinflammatory or anti-inflammatory effects on vessels and other tissues [21]. It is possible that *Tff3^−/−^* mice, due to changes of lipid metabolism, have changes in the balance of vasoactive prostaglandins, which could affect their vascular reactivity to stimuli such as FID and may also affect the ability of neuronal tissue to sustain ischemia in stroke. Since inflammation is part of the tissue damage, changes in the proinflammatory and anti-inflammatory factors could also be beneficial in the outcomes of stroke.

The aim of the present study was to evaluate the effects of one week of HS diet on endothelium-dependent responses to flow-induced dilation (FID) and acetylcholine (ACh; 10^−6^ M) and endothelium-independent response to the NO donor sodium nitroprusside (SNP; 10^−6^ M) in isolated, pressurized carotid arteries of *Tff3^−/−^*/C57BL/6N (*Tff3^−/−^)* mice and their wild-type controls, C57BL/6N mice (WT). To further evaluate to role of NO in vascular relaxation responses, we assessed vascular production of NO by direct fluorescence.

Accumulation of misfolded proteins in cells triggers the activation of the unfolded protein response (UPR). Unresolved ER stress underlies the development of various diseases [22] and since it has been shown that the Tff3 protein can interact with proteins participating in the UPR, we have monitored relevant ER stress markers. Furthermore, ischemic injury of heart [23] and brain causes Tff3 upregulation in liver from where it is released into the circulation, and can access injured tissues and act protectively. In addition to ERS markers, we analyzed the expression of genes involved in inflammation pathways of oxidative stress that are relevant in different pathological conditions.

Tff3 has been shown to be cytoprotective in the gut. Zhang et al. [24] demonstrated the significant impact of recombinant TFF3 protein on hypoxia-induced necrotizing enterocolitis in immature rats. The tissue level of interleukin-8, tumor necrosis factor-alpha, malondialdehyde, prostaglandin E_2_, tromboxane B_2_, and nitric oxide were significantly decreased in treated mice compared to the mice without treatment, and inducible nitric oxide synthase and cyclooxygenase 2 were also decreased, suggesting an important regulatory role of Tff3 in the expression of these molecules in their model. However, there is a lack of data elucidating the regulatory or functional role of the *Tff3* gene or Tff3 protein in the mechanism of vascular reactivity. Also, Tff3 protein deficiency in circulation could affect the structural properties of blood vessels. The functional and structural properties of arteries and the effect of HS diet/genotype interactions were assessed with proteomic analysis and histological analysis of carotid tissue, respectively. In addition, to evaluate the functional effects of dietary/genetic alterations, we examined the effect of HS intake on the cerebral infarct volume in *Tff3^−/−^* animals compared to their WT control group.

## 2. Results

### 2.1. Flow-Induced Relaxation of Carotid Arteries

**Figure 1:**
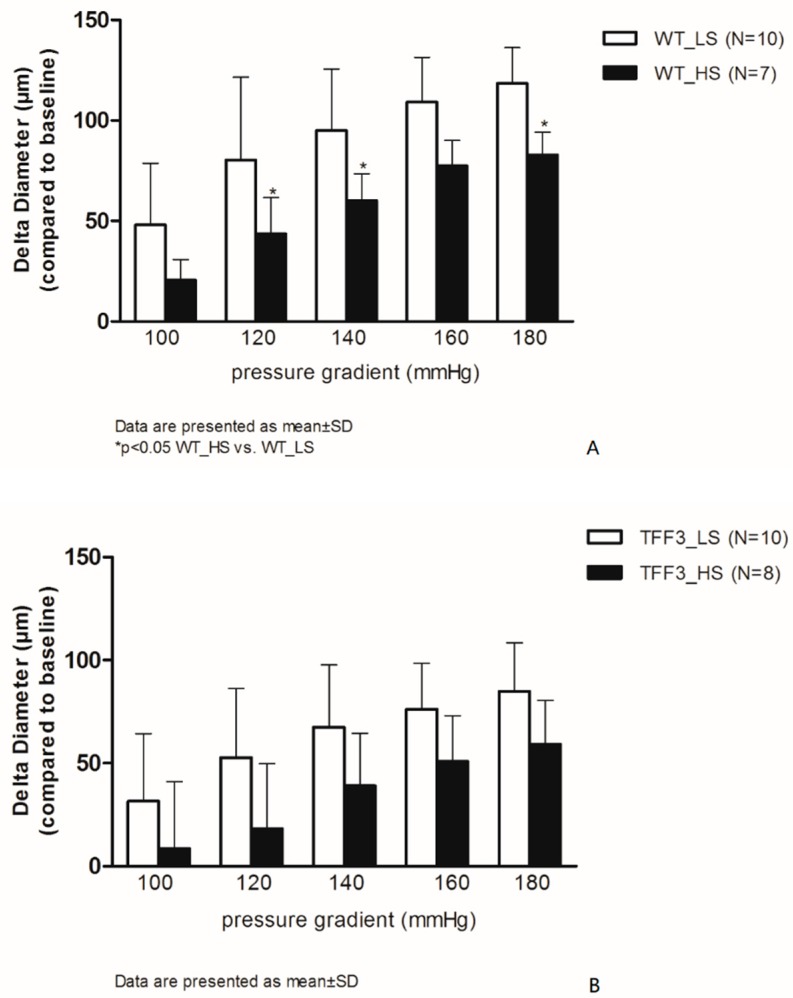
Flow-induced relaxation (FIR) of carotid arteries in wild-type (WT) (**A**) and TFF3 (**B**) groups of mice on low salt (LS) and high salt (HS) diet. The differences in dilatation is expressed as delta diameter (μm) compared to baseline (no flow condition, 0 mmHg) of carotid arteries between LS (*N* = 10) and HS (*N* = 7) groups of animals in response to stepwise increases in pressure gradient (100–180 mmHg). Data are presented as mean ±SD. * Significant difference (*p* < 0.05) between LS and HS groups.

Figure 1 summarizes the flow-induced relaxation (FIR) responses of carotid arteries in the WT group of mice, baseline (LS), and after high salt intake (HS) (panel A). One week of HS diet significantly reduces FIR at ∆120, 140, and 180 mmHg in the WT HS group of mice compared to WT LS group. In the*Tff3^−/−^* groups of animals (TFF3_LS and TFF3_HS), the FIR was similar between the LS and HS groups (Figure 1B). There were no statistically significant differences in vasodilation between WT and *Tff3*^−/−^ mice on LS diet or between the WT-HS group and the *Tff3^−/−^-*HS group of mice.

**Figure 2:**
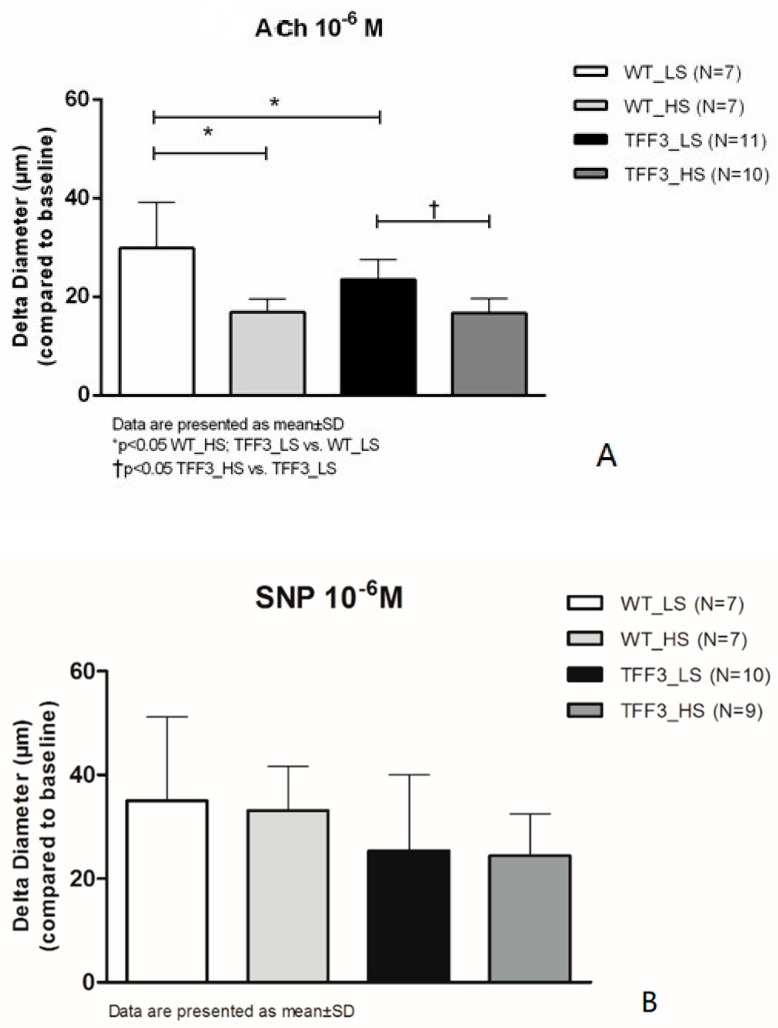
Relaxation of carotid arteries in response to ACh (**A**) and Sodium-Nitroprusside (SNP; **B**) in WT and TFF3 mice on LS and HS diet. The differences in responses to acetylcholine (10^−6^ mol/L) is expressed as delta diameter (μm) compared to baseline (no flow condition, 0 mmHg) of carotid arteries between WT (*N* = 7) and TFF3 (*N* = 11) groups of animals fed with standard chow (0.4% NaCl) for one week, and WT (*N* = 7) and TFF3 (*N* = 10) groups of animals fed with food containing 4% NaCl (HS) for one week. Data are presented as mean ±SD. * Significant difference (*p* < 0.05).

ACh-induced dilation was significantly decreased in *Tff3^−/−^* LS mice compared to WT LS mice. High salt intake for one week reduced the ACh-induced dilatation in WT group and in *Tff3^−/−^* group of mice compared to respective LS control groups (Figure 2A), while vasodilation in response to SNP was preserved at all study groups, and there was no significant difference in the response among the groups (Figure 2B).

### 2.2. In Situ Evaluation of Nitric Oxide (NO) Levels in Carotid Arteries by Fluorescence Microscopy

**Figure 3:**
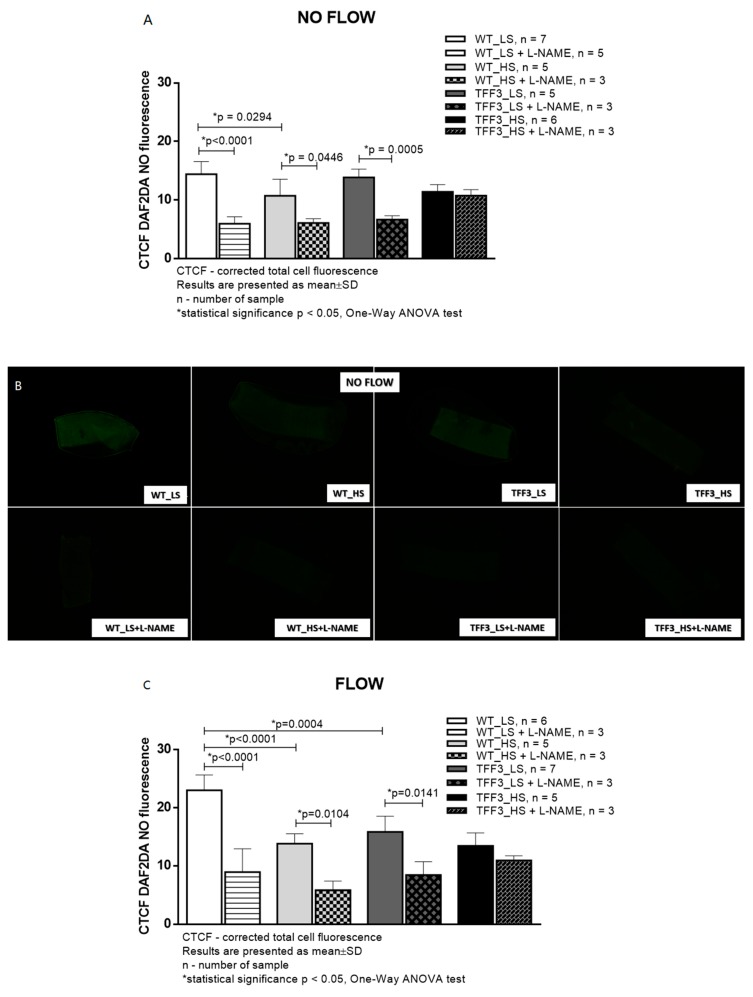

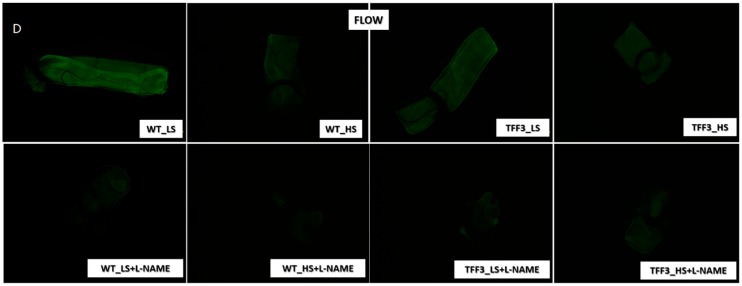
Nitric oxide levels (**B** and **D**) in carotid arteries of wild type (WT) and *Tff3-*depleted mice on normal (LS) and high salt diet (HS) assessed by fluorescence without (**A** and **B**) and with (**C** and **D**) flow through the artery. CTCF—corrected total cell fluorescence; HS—high-salt diet; NS—normal-salt diet; L-NAME—N^ω^-nitro-L-arginine methyl ester, Objective: 5×; scale bar 200 μm, * Significant difference (*p* < 0.05).

Basal (no-flow) production of NO was significantly decreased in WT HS mice compared to WT LS mice. Other groups exhibited similar levels of NO production under no-flow conditions. L-NAME significantly decreased NO production in all groups of mice, except in *Tff3^−/−^* HS mice. Under flow conditions, NO production in carotid arteries of WT HS mice was significantly lower than in WT LS mice. Flow-induced NO production in *Tff3^−/−^* LS knockout mice was significantly lower than flow-induced NO production in WT LS mice. Flow-induced NO production in *Tff3^−/−^* HS mice was similar to *Tff3^−/−^* LS mice NO production, and to both WT groups. L-NAME significantly decreased NO production in these three groups of mice, and did not affect flow-induced NO production in carotid arteries of *Tff3^−/−^* HS mice (Figure 3).

### 2.3. Biochemical Analysis of the Sera

Serum levels of measured biochemical parameters are presented in Table 1. None of the parameters were affected either by HS or the knock down of the *Tff3* gene.

### 2.4. Proteomic Analysis Revealed a Significant Effect of Tff3 Gene Deficiency on the HS Diet Induced Changes in Protein Expression in Carotid Arteries

**Figure 4:**
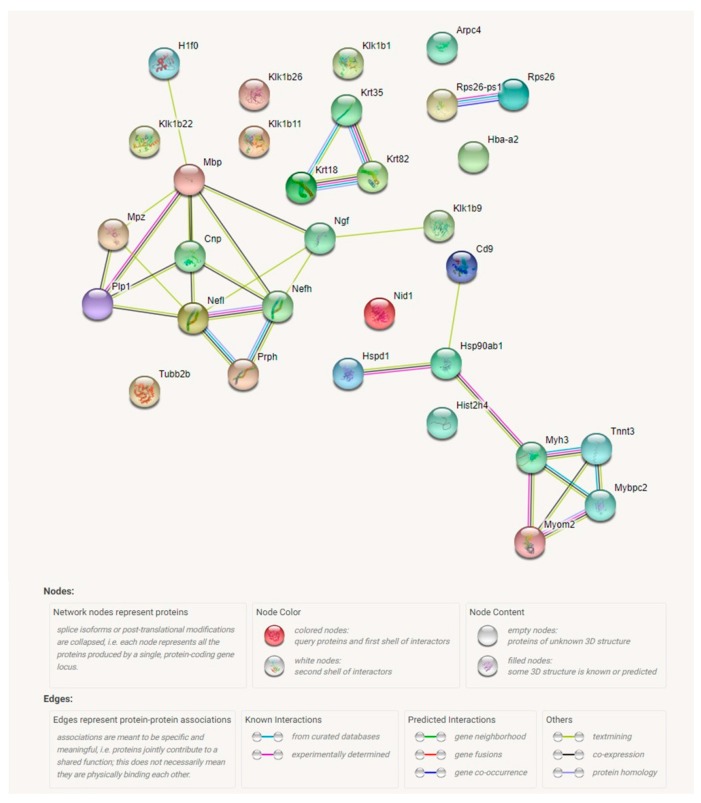
Schematic representation of protein interaction networks calculated for proteins that are differentially expressed in carotid arteries of *Tff3^−/−^* mice on high salt diet (HS) compared to WT mice on a HS. Interactions were visualized by STRING program. (https://string-db.org/cgi/ntwork.pl?taskId=g1if05lDMz7j). Arpc4—Actin-related protein 2/3 complex subunit 4; Cd9—CD9 antigen; Cnp—2′,3′-cyclic-nucleotide 3′-phosphodiesterase; H1f0—Histone H1.0; Hba-a2—Hemoglobin alpha, adult chain 2; Hist2h4—Histone cluster 2; Hsp90ab1—Heat shock protein HSP 90-beta; Hspd1—60 kDa heat shock protein, mitochondrial; Klk1b1—Kallikrein 1-related peptidase b1; Klk1b11—Kallikrein 1-related peptidase b11; Klk1b22—Kallikrein 1-related peptidase b22; Klk1b26—Kallikrein 1-related peptidase b26; Klk1b9—Kallikrein 1-related peptidase b9; Krt18—Keratin, type I cytoskeletal 18; Krt35—Keratin type I; Krt82—Keratin type II, cuticular Hb2 (Keratin 82); Mbp—Myelin basic protein; Mpz—Myelin protein P0; Mybpc2—Myosin-binding protein C, fast-type; Myh3—Myosin-3; Myom2—Myomesin 2; Nefh—Neurofilament heavy polypeptide; Nefl—Neurofilament light polypeptide; Ngf—Beta-nerve growth factor; Nid1—Nidogen-1; Plp1—Myelin proteolipid protein; Prph—Peripherin; Rps26—Ribosomal protein S26; Rps26-ps1—Ribosomal protein S26, pseudogene 1; Tnnt3—Troponin T, fast skeletal muscle; Tubb2b—Tubulin beta-2B chain.

Results of proteomic analysis of the differentially expressed proteins in carotid arteries of Tff3−/− mice on high salt diet (HS) compared to WT mice on HS diet are presented in Table 2. Figure 4 presents the proteomic analysis of carotid arteries. Proteins isolated from carotid arteries of *Tff3^−/−^* mice or WT controls on a HS diet were analyzed by a quantitative Tandem Mass Tags approach using a Q-Exactive-Plus mass-spectrometer. As a result, 3900 quantifiable proteins were identified using Proteome Discoverer. Initially, 186 significantly regulated proteins were found, including proteins involved in cell organization and biogenesis, cell proliferation, cell death, and metabolic processes. Moreover, some of those proteins are involved in several different signaling pathways, such as SIDS susceptibility pathways, MAPK signaling pathway, EGFR1 signaling pathway, TNF-alpha and NF-κB signaling pathway; focal adhesion-PI3K-Akt-mTOR-signaling pathway, and type II interferon signaling (IFNG). Abundance ratio of protein expression was in the range from 0.221 (Keratin 35) to 9.625 (heat shock protein 65). Statistically relevant proteins with 50% increased/decreased expression were extracted (40 different entities) for identification of protein networks using STRING (Figure 4 and Supplementary Figure S1). This analysis revealed five networks, including (1) interaction of three structural proteins keratin 18, keratin 35, and keratin 82; (2) a group of structural and functional neuronal proteins (Neurofilament heavy polypeptide, kallikrein 1-related peptidase b22, 2′,3′-cyclic-nucleotide 3′-phosphodiesterase, myelin protein P0, myelin proteolipid protein, neurofilament heavy polypeptide); (3) proteins involved in innate immune system activation, and TNF-alpha and NF-κB signaling (heat shock protein HSP 90-beta, 60 kDa heat shock protein, CD9 antigen ad Histone cluster 2, H4) which is in direct interaction with (4) a group of structural and functional/contraction muscle cell related proteins (myosin-binding protein C, fast-type, myomesin 2, myosin-3, Troponin T) and a cluster of two ribosomal proteins (Ribosomal protein S26, Ribosomal protein S26, pseudogene 1).

### 2.5. Expression Levels of Genes Encoding for ER Stress Markers, Oxidative Stress, and Cytokines

Expression levels of genes encoding ER stress markers, oxidative stress/antioxidative enzymes and proinflammatory cytokines were determined in liver tissue from *Tff3^−/−^* and WT animals exposed to HS diet or LS diet during brain ischemia protocol.

**Figure 5:**
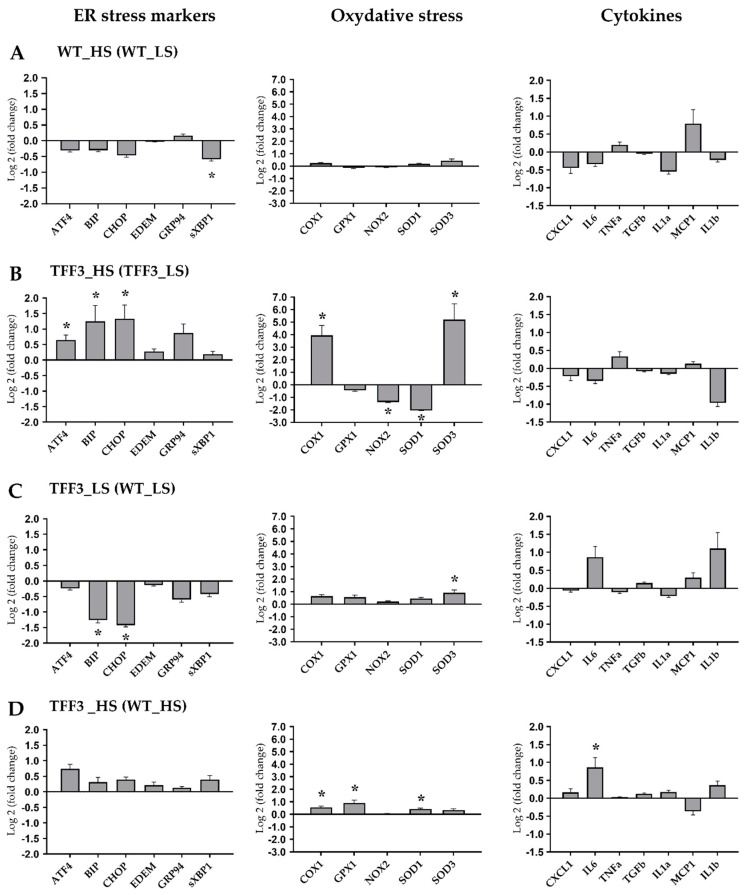
Expression levels of genes encoding for ER stress markers, oxidative stress/antioxidative enzymes and proinflammatory cytokines were determined in liver tissue from *Tff3^−/−^* and WT animals exposed to high salt diet (HS; 4% NaCl) or LS (0.4% NaCl) *ad libitum* (*N* = 4–6 animals per group). qPCR was performed using Sybrgreen detection and Ct data were analyzed by REST program. Data are expressed as log2 of fold change for WT HS group relative to WT LS (**A**), *Tff3^−/−^* HS group relative to *Tff3^−/−^* LS (**B**), *Tff3^−/−^* LS group relative to WT LS (**C**) and *Tff3^−/−^* HS group relative to WT HS (**D**). Data were compared as described in order to assess the effects of the diet (**A** and **B**) or the genotype (**C** and **D**). * Significant difference (*p* < 0.05).

In WT mice, short term HS diet resulted in significant downregulation of sXBP1 gene relatively to WT LS group (1.51-fold; *p =* 0.036), while there was no significant difference in expression levels of remaining ER stress markers, antioxidative enzymes and cytokines genes (Figure 5A).

Contrary to WT mice, the *Tff3* deficient mice on HS (TFF3 HS group) had significantly higher mRNA levels of *ATF4* (1.57-fold; *p =* 0.038), *BIP* (2.38-fold; *p =* 0.049), *CHOP* (2.51-fold; *p =* 0.016), *COX1* (15.4-fold; *p =* 0.002) and *SOD3* (36-fold; *p =* 0.002) genes, while *NOX2* (2.56-fold; *p =* 0.001) and *SOD1* (4.12-fold; *p =* 0.001) were significantly downregulated relatively in *Tff3^−/−^* HS group (Figure 5B).

The sole knockout of *Tff3* gene in animals on LS diet resulted in downregulation of *BIP* (2.40-fold; *p =* 0.044) and *CHOP* (2.68-fold; *p =* 0.012) genes, and an approximately two-fold upregulation of the *SOD1* gene (*p =* 0.042; Figure 5C).

Finally, we assessed the influence of *Tff3* gene knockout on the effects of HS by comparing *Tff3^−/−^* HS group of mice relatively to WT HS group. *Tff3^−/−^* HS mice had significantly higher mRNA levels of *COX1* (1.47-fold; *p =* 0.046), *GPX1* (1.88-fold; *p =* 0.021), *SOD1* (1.35-fold; *p =* 0.015) and *IL-6* genes (1.81-fold; *p =* 0.05; Figure 5D).

Taken together, HS had only a minor effect on target genes’ expression in WT mice (Figure 5A, which is in contrast to *Tff3^−/−^* mice fed HS that showed differential expression of several target genes, including a 15- and 36-fold increased mRNA expression levels of *COX1* and *SOD3* genes, respectively (Figure 5B).

*Tff3* deficient mice have different response on HS diet compared to *Tff3* deficient^-^ mice on a LS diet in terms of ER stress relevant genes and oxidative stress genes (Figure 5A,B). Animals exposed to stroke showed that *Tff3* deficient mice on a LS diet had more pronounced brain infarct volume. The liver of these mice showed significant downregulation of ER stress relevant genes (*BIP*, *CHOP*; Figure 5C) compared to WT mice. The liver of HS diet fed *Tff3* deficient mice showed no difference in activation of ER stress relevant markers compared to WT mice.

### 2.6. Activation of ER Stress Relevant eIF2α Protein

**Figure 6:**
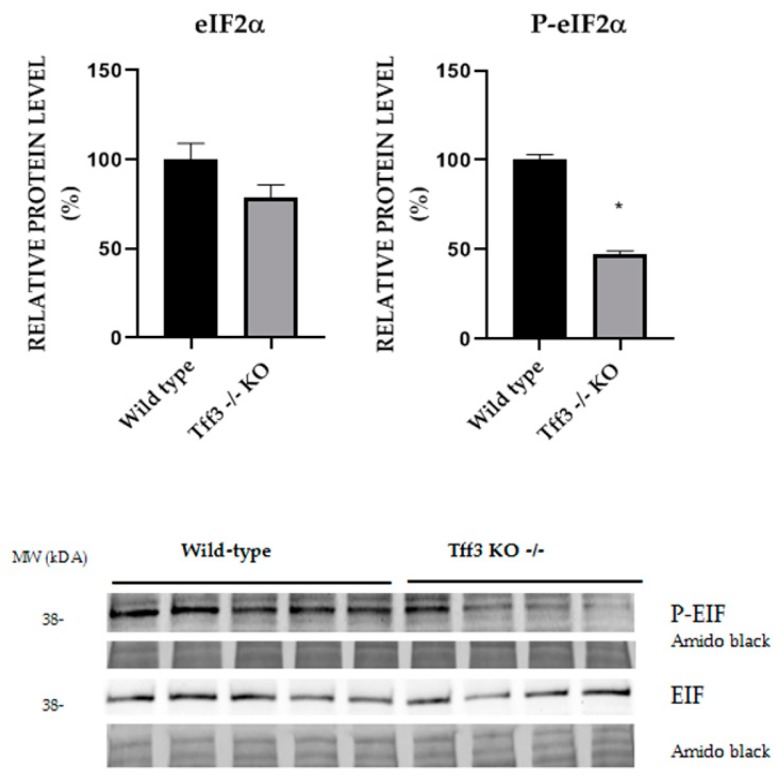
Effect of ER stress on eIF2α protein phosphorylation. Relative protein level of ER stress markers eIF2α and p-eIF2α in liver homogenate of WT LS mice and *Tff3^−/−^* LS mice with induced stroke. Protein level is presented relative to WT mice as mean ± SEM of specific protein band density normalized with amido black. The difference between groups was compared by Student *t*-test. * Significant difference (*p* < 0.05).

We compared the level of eIF2α activation in liver of WT and *Tff3^−/−^* mice on LS diet which showed difference in stroke intensity (Figure 6). *Tff3* deficient mice have 50% reduced activation of P-eIF2*α*.

### 2.7. Structural Characteristics of Aortas of Tff3^−/−^ and WT Mice on LS and HS Diet

**Figure 7:**
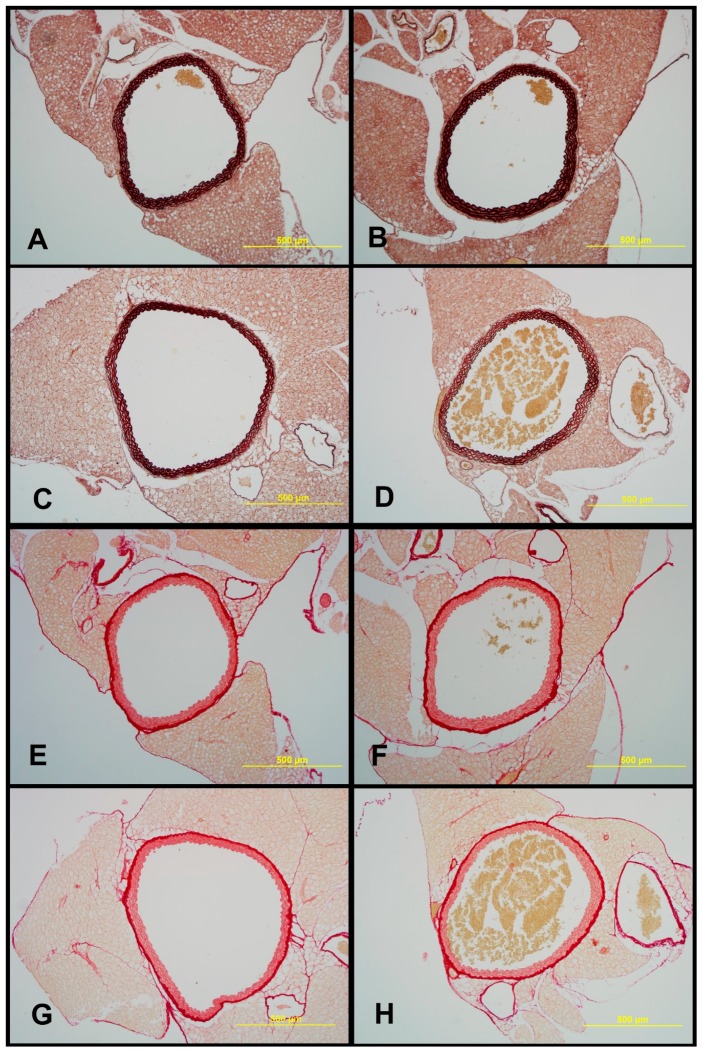
Sections of aortae representing each experimental group: WT LS (**A** and **E**), WT HS (**B** and **F**), *Tff3^−/−^* LS (**C** and **G**), *Tff3^−/−^* HS (**D** and **H**). Sections were stained with orcein for visualizing elastin content (**A**–**D**) and picrosirius red for visualizing collagen fibers (**E**–**H**). Objective: 10×; scale bar: 500 μm.

**Figure 8:**
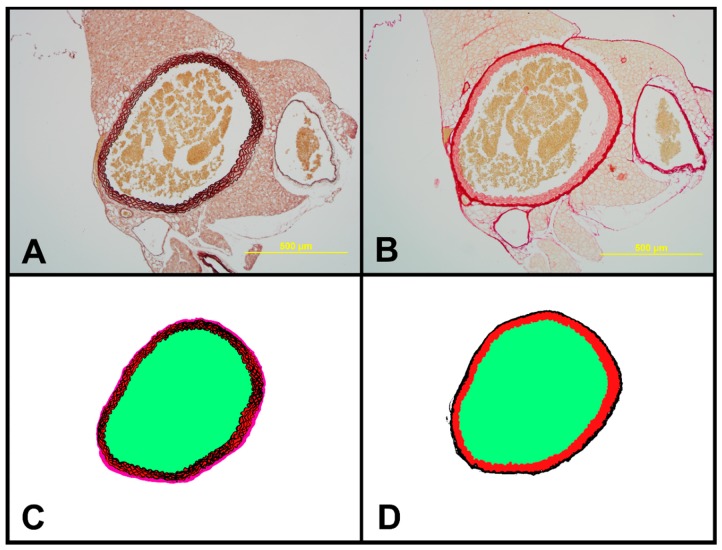
Representative sections of aorta of *Tff3^−/−^* mice on HS used for histomorphological analysis. (**A**) Orcein-stained aorta with elastic fibers stained black to brown. Objective 10×, scale bar 500 μm. (**B**) Picrosirius red-stained aorta showing amount of collagen (red). Objective 10×, scale bar 500 μm. (**C**) Color-coded segmented mask from orcein-stained section. Elastic fibers are shown in black, tunica media in red, tunica adventitia in pink, and lumen area in green. (**D**) Color-coded segmented mask from picrosirius red-stained section. Collagen is shown in black, tunica media in red, and lumen area in green.

Morphological analysis under light microscope revealed normal structure of all layers of aorta in all examined groups. Tunica media was the thickest layer, containing 5–6 elastic laminas, including the internal elastic membrane (Figure 7 and Figure 8). *Tff3^−/−^* mice on a LS diet had significantly higher elastin content and media thickness and significantly decreased adventitia and wall to lumen ratio compared to WT LS controls. Aortas of WT HS had significantly increased elastin content and WT HS mice and *Tff3^−/−^* HS mice had significantly increased media thickness and decreased collagen component compared to WT LS mice. There were no observed differences in the aorta structure between *Tff3^−/−^* LS and *Tff3^−/−^* HS groups of mice (Table 3).

### 2.8. Effects of Dietary Protocol and Strain Difference on the Brain Infarct Volume in Tff3^−/−^ and WT Mice

**Figure 9:**
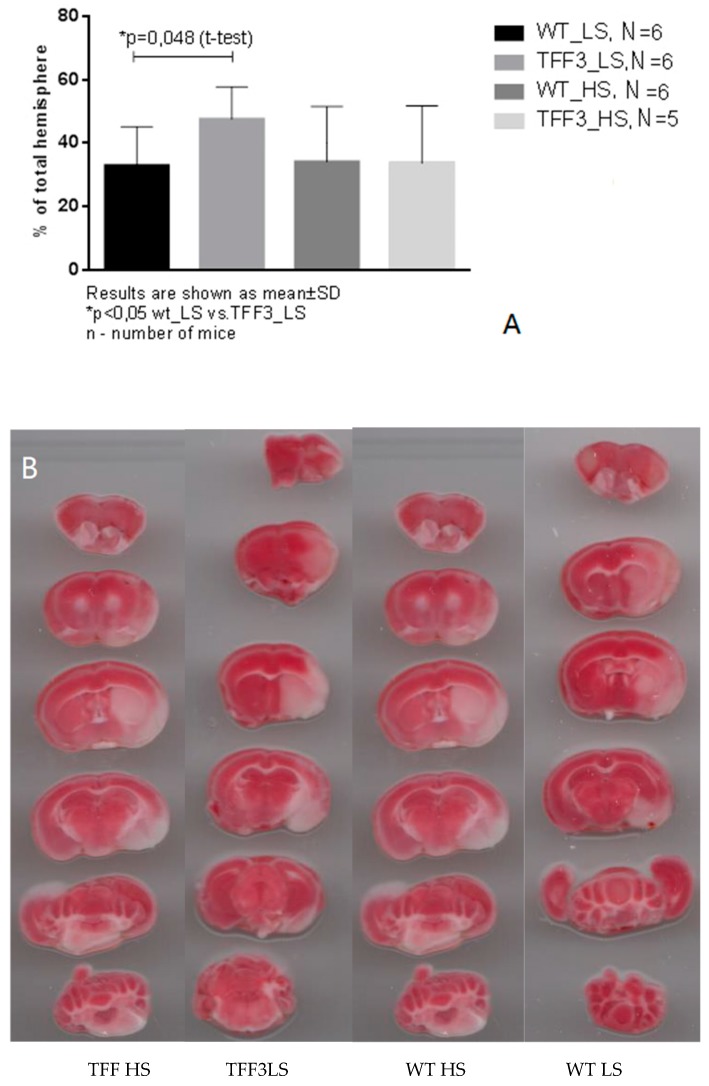
(**A**) Brain infarction size in the TFF3^−/−^ groups and WT groups on HS diet and LS diet (Tff3^−/−^ HS *N* = 5, Tff3^−/−^ LS *N* = 6, WT HS *N* = 6, WT LS *N* = 6). **(B)** Typical brain infarction in groups after 60 min of transitory middle cerebral artery occlusion (toMCAO) in all experimental groups. In all groups, toMCAO induced cerebral infarction. Sections were stained with 2,3,5-triphenyltetrazolium chloride (TTC). Healthy tissue is of brick-red color, while the infarcted tissue, lacking the mitochondrial enzymes necessary for the transformation of TTC, remains white.

In all groups, toMCAO induced cerebral infarction; however, significant difference was only observed in the TFF3^−/−^ LS group where toMCAO produced significantly larger infarction compared to WT LS mice (Figure 9A). HS diet-fed *Tff3* deficient mice seem to be less susceptible to stroke injury (Figure 9).

## 3. Discussion

The salient findings of the present study are: (a) High salt diet significantly altered endothelium-dependent, but not independent, vasorelaxation of carotid arteries in WT mice and partially in *Tff3^−/−^* mice. This was underlined by decreased vascular production of NO (as revealed by direct fluorescence assay); (b) Additionally, structural analysis revealed that HS diet did not affect structural properties of the *Tff3^−/−^* mice aorta. However, there is a significant effect of the HS diet on the structure of WT mice aortas, suggesting a protective effect of *Tff3^−/−^* depletion on vasculature; (c) carotid artery proteome analysis demonstrated a very complex interaction of gene depletion and diet, involving networks of structural, smooth muscle-related, and structural neuronal proteins, and innate immunity and inflammatory. (d) Interestingly, stroke was similar in both groups of animals on HS diet, suggesting that HS diet did not aggravate the effects of ischemia in both experimental groups. However, *Tff3^−/−^* LS mice exhibited bigger strokes compared to WT LS mice, possibly due to their decreased ER stress response.

### 3.1. High Dietary Salt Intake and Vascular Function

High salt (HS) dietary intake represents a worldwide health problem [3,25]. A recent study has demonstrated that most populations have a mean sodium intake in excess of 100 mmol/d (2.30 g/d), and in many (especially the Asian countries), in excess of 200 mmol/d (4.60 g/d) [26]. This is associated with increased blood pressure and is one of the most important risk factors for cerebrovascular incidents [27]. High salt intake is related to impaired vascular function, e.g., affecting the mechanisms of endothelium dependent vasodilation, increased level of oxidative stress, decreased NO bioavailability, and shifted production and sensitivity to metabolites of arachidonic acid, as demonstrated in animal models and in humans [5,6,7,28].

Genetically altered rodents (e.g., consomic and congenic rats and various models of transgenic mice) are commonly used in assessment of mechanisms of vascular function and stroke [15,29]. In the present study, we have utilized a novel model for vascular studies, *Tff3^−/−^* mice. The Tff3 protein belongs to the trefoil factor family (TFF) of peptides that were first recognized in the GI tract where they have an important role in the maintenance of epithelial integrity and, in the case of injury, in promoting tissue repair [30,31]. In recent years, they have been identified in tissues other than GI, with emerging (patho)physiological functions in tumor progression, angiogenesis, and immunity [32]. Herein, we demonstrated for the first time that *Tff3*-depleted mice and their WT control could be utilized for cerebrovascular studies due to their unique phenotype [19] and potential implications on vasoreactivity and inflammatory response to environmental stressors, such as high salt diet.

### 3.2. Vascular Relaxation Responses and the Role of NO in Flow-Induced Relaxation of Carotid Arteries

Playing a pivotal role in the maintenance of basal vascular tone [33], NO also has a central role in vasodilatation [34], preventing the adhesion and migration of leukocytes into the arterial wall and inhibiting the proliferation of vascular smooth muscle cells [35]. Shear force is the main stimulator of NO production by phosphorylation of endothelial NO synthase (eNOS) and tyrosine kinases, which regulate endothelial NO production through regulation of eNOS [36]. In addition, ACh, bradykinin, thrombin, adenosine diphosphate (ADP), and adenosine triphosphate (ATP) relax vascular smooth muscle cells and stimulate production of NO [35,37,38]. Our results confirmed that the endothelium-dependent vasodilatation in response to ACh was reduced significantly after high salt intake in both strains, while FID was significantly affected by HS diet only in WT mice, suggesting partially modified mechanisms of endothelium-dependent responses in *Tff3^−/−^* mice. FID has been demonstrated to be mediated by NO, endothelium-derived relaxing factor (EDRF), prostacyclin (PGI_2_), and, paradoxically, by endothelium-derived contracting factors (EDCF), e.g., endothelin (ET-1), thromboxane A_2_ (TXA_2_), and modified by excess of reactive oxygen species (ROS) [39,40], In our recent study, HS diet shifted the mechanisms of FID from NO-, prostanoid-, and EETs-mediated toward a blunted and solely NO-mediated response [5]. In the present study, these observations of functional responses are further supported by direct fluorescence assessment of vascular NO production, demonstrating that a HS diet significantly decreased the flow-induced NO production in WT mice. Vasodilatation in response to SNP was preserved at all study groups and there was no significant difference in the response between the groups (Figure 2), suggesting preserved reactivity of vascular smooth muscle.

### 3.3. Biochemical Analysis of the Sera

None of the serum levels of measured biochemical parameters were affected either by HS or the knock down of the *Tff3* gene (Table 1). Similar results were obtained in study by Bujak et al., for *Tff3-*depleted mice and their controls on a regular standard rodent chow [19].

### 3.4. Salt Intake and Tff3 Gene Depletion Affect the Structural Components of the Vessel

Ever since the discovery of Tff proteins, they have been subject to research primarily in the context of physiology and pathology of different mucous membranes. Nevertheless, recent research investigated the role of Tff3 peptide in several connective tissues. Rösler et al., have showed that Tff3 peptide affects matrix metalloproteinases in cartilage and hence influences cartilage matrix remodeling in inflammatory cartilage diseases [41]. Furthermore, Tff3 peptide has been discovered in the process of endochondral ossification in mouse fetuses, and *Tff3* knock-out mice have lower quality of cancellous bone than wild-type mice [21,42,43]. Tff3 displays a proangiogenic activity, since it promotes formation of capillary vessels in chorioallantoic membrane (CAM) assay. The number of microvessels in breast and gastric cancer correlates significantly with Tff3 expression [30,44]. According to Lau et al., Tff3 expressed in breast cancer cells promotes de novo angiogenesis directly through stimulation of endothelial cells and indirectly through increased expression of IL-8 [45]. However, no Tff3 expression has been described in large blood vessels, such as aorta [46].

In the present study, some structural differences in the analyzed components of vessels were observed between strains, i.e., higher elastin content and media thickness and significantly decreased adventitia and wall to lumen ratio in *Tff3^−/−^* LS mice compared to WT LS controls. High salt diet affected vascular structure mainly in WT groups of mice, resulting in increased elastin % in WT HS mice compared to WT LS mice. *Tff3* gene depletion seems to protect from dietary induced structural vascular changes, since no differences in the aortic structural components (elastin or collagen) or structural remodeling between *Tff3^−/−^* LS and *Tff3^−/−^* HS groups of mice were observed (Table 3). Interestingly, *Tff3* gene deficiency exhibited a significant effect on the HS diet-induced changes in protein expression in carotid arteries, which was revealed by proteomic analysis (Table 2, Figure 4). Potentially interesting networks that may affect vascular function are the interaction of three structural proteins, keratin 18, keratin 35, and keratin 82, which were mostly downregulated with HS diet; and a group of structural and functional/contraction muscle cell related proteins (myosin-binding protein C, fast-type, myomesin 2, myosin-3, Troponin T) which were upregulated with HS diet. However, this finding requires further exploration and analysis in terms of their functional significance.

### 3.5. The Effects of Diet and Gene Depletion on Systemic Inflammatory Response and Oxidative Stress

Nowadays, it is generally accepted that low grade inflammation is present in vascular tissue in all cardiometabolic diseases (e.g., hypertension, diabetes mellitus, atherosclerosis) [47]. Furthermore, oxidative stress and inflammation underlies neural tissue ischemia-reperfusion injury. High salt dietary intake impairs vascular function via endothelial activation [5,6,7]. The liver is the source of the proteins of acute phase inflammatory response, such as TNFa, IL-1 and IL6. They are involved in a systemic reaction of the organism to infection, tissue injury, trauma or surgery, neoplastic growth or immunological disorders known as the acute phase response [48]. The reaction involves release of proinflammatory cytokine, activation of immune cells and vascular system. High salt diet represents environmental stressor which could affect native immune system activation. Results of the present study suggest that HS had only a minor effect on target gene expression in WT mice (Figure 5 A, which is in contrast to *Tff3-*depleted mice fed HS that showed differential expression of several target genes, including a 15- and 36-fold increased mRNA expression levels of *COX1* and *SOD3* genes, respectively (Figure 5B). The response to high salt diet was affected by *Tff3* gene depletion. HS diet upregulated *GPX1* (1.88-fold; *p =* 0.021), *SOD1* (1.35-fold; *p =* 0.015), and *IL-6* genes (1.81-fold; *p =* 0.05; Figure 5D) in *Tff3*-depleted mice compared to WT HS controls. It has been observed that increased levels of NaCl may increase in intracellular and surface expression of HSP60 protein in human umbilical vein endothelial cells. Additionally, the increased Na may induce cellular apoptosis [49]. Taken together, our results suggest that antioxidative system has been activated in *Tff3-*depleted mice in response to HS diet, presumably contributing to their lower level of inflammation and better vascular functional response and fact that they have been protected from neural tissue damage (as demonstrated in our stroke experiments, Figure 9A).

### 3.6. Tff3 Gene Depletion, but not Short-Term High Salt Diet Increases the Stroke Volume; the Role of Proteome Modification

Short-term and chronic HS diets lead to impaired autoregulation in the cerebral circulation, as demonstrated in Sprague–Dawley rats [50]. Common carotid artery is effective “pressure dampener” along its length, and contributes to a significant reduction in pulse pressure [51], thus protecting cerebral microcirculation. Carotid-cerebral pulse wave velocity (ccPWV) reflects the segment (C-M segment) stiffness between the common carotid artery and ipsilateral middle cerebral artery [51]. Recently, it has been shown that C-M segment atherosclerosis was independently associated with higher systolic blood pressure, ccPWV, and carotid intima-media thickness in eighty-one acute ischemic stroke patients [52]. Taken together, these results support the importance of elucidating vascular reactivity mechanisms in conductive arteries, such as carotid artery.

Data on the Tff3 gene/protein role in cerebral ischemia-reperfusion injury are scarce. For example, a recent study demonstrated that following the myocardial [53] and brain ischemia, mice unable to synthesize adequate quantities of the Tff3 protein in their liver lack the protective effect of the Tff3 protein in serum, resulting in greater tissue damage. Thus, such mice exhibited significantly higher activity of caspase 3 and consequentially a higher level of cell death in the ischemic cerebral lesion, together with a larger fraction of cerebral infarcts and a smaller fraction of the injured cerebral hemisphere. They had more prominent forelimb motor deficits. Recombinant Tff3 protein administered intravenously reversed the cerebral injury and forelimb motor function, indicating that an endocrine neuroprotective mechanism that uses liver Tff3 protein is engaged in cerebral ischemia/reperfusion injury [54]. Our results are in agreement with these observations by Liu et al., In the present study, *Tff3^−/−^* LS mice exhibited bigger cerebral infarction compared to WT LS mice. Furthermore, in the present study, proteomic analysis demonstrated significant changes in a group of structural and functional neuronal proteins (neurofilament heavy polypeptide, kallikrein 1-related peptidase b22, 2′,3′-cyclic-nucleotide 3′-phosphodiesterase, myelin protein P0, myelin proteolipid protein, neurofilament heavy polypeptide); and proteins involved in innate immune system activation, and TNF-alpha and NF-κB signaling (heat shock protein HSP 90-beta, 60 kDa heat shock protein, CD9 antigen ad Histone cluster 2, H4) in *Tff3^−/−^* HS mice compared to WT HS mice. However, the significance of observed changes in protein expression remains to be established. It seems that short-term high salt diet has not augmented the stroke in *Tff3^−/−^* HS mice, because the infarction was similar between *Tff3^−/−^* LS and *Tff3^−/−^* HS mice (Figure 9A), possibly due to short duration of the HS diet. Recently, it has been suggested that administration of arachidonic acid could inhibit inflammatory response and oxidative stress, thus attenuating brain tissue damage in the rat model of ischemia-reperfusion injury [55]. In the present study, analysis of ER stress markers eIF2α and p-eIF2α in liver homogenate of WT LS mice and *Tff3^−/−^* LS mice with induced stroke demonstrated that *Tff3*-deficient mice on LS diet had 50% lower activation of p-eIF2α compared to WT controls on a LS diet, suggesting a downregulated ERS response. These results are in line with *CHOP* and *BIP* downregulation shown at the transcription level (Figure 5C). eIF2α is a downstream target of PERK activation, as well as CHOP, a transcriptional factor with a proapoptotic role. Unfolded or misfolded protein accumulation causes endoplasmic reticulum chaperone BIP (BIP) to dissociate from PKR-like ER kinase (PERK), one of three main UPR. PERK phosphorylates and consequently activates EIF2α, a subunit of the ribosome, which results in activation of transcriptional factors, including CHOP [56]. Prolonged stress leads to the activation of proapoptotic pathways. Our results indicate that *Tff3* depletion led to diminished ER stress response, specifically the PERK pathway.

## 4. Materials and Methods

### 4.1. Dietary Protocols

Ten-week-old transgenic *Tff3^−/−^/*C57BL/6N (*Tff3^−/−^)* knockout mice and WT/C57BL/6N (WT) (parental strain) healthy male mice were divided in LS group and HS group and fed for one week with standard rodent chow (0.4% NaCl; LS group) and food containing 4% NaCl (HS group), respectively and let to drink water *ad libitum*. All of the above experimental procedures were in line with the European Guidelines for the Care and Use of Laboratory Animals (Directive 86/609), as well as approved by the local Ethical Committee (#2158-61-07-18-138) on 28 September 2018., National Ethical Committee (EP 195/2019) and Ministry of Agriculture (#525-10/0255-19-7) on 12 April 2019.

### 4.2. Endothelium-Dependent and Endothelium-Independent Relaxation Responses of Carotid Arteries of TFF3 and WT Mice on LS and HS Diet

#### Isolated Carotid Artery Protocols

The mice were anesthetized with ketamine chloride (100 mg/kg) and midazolam (5 mg/kg), weighed, and their carotid arteries were isolated. Afterwards, mice were decapitated. The carotid arteries were cannulated on glass micropipettes in a chamber filled with isotonic physiological salt solution (PSS) warmed to 37 °C and oxygenated with 21% O_2_–5% CO_2_–balance N_2_. The PSS used in these experiments contained (in mmol/L) 119 NaCl, 4.7 KCl,1.17 MgSO_4_, 1.6 CaCl_2_, 1.18NaH_2_PO_4_, 24 NaHCO_3_, 0.026 EDTA, and 5.5 glucose. EDTA was purchased from Sigma-Aldrich, CaCl_2_·2H_2_O and NaHCO_3_ were purchased from Merck, while the rest of the chemicals were purchased from Kemika (Zagreb, Croatia) [7]. After 60 min of incubation at intraluminal pressure of 100 mmHg (equilibration time) to assess their basal diameter (basic response), baseline diameter was then determined by measuring the diameter of the arteries pressurized at 80 mmHg and without flow. The arteries were subjected to flow at pressure gradients from Δ10–Δ180 mmHg (flow-induced responses; FIR) (Myograph Pressure System Model 110P MyoView Version 1.2.0 DMT, Danish Myo Technology, Hinnerup, Denmark For each pressure gradient (10–180 mmHg), the level of dilatation was expressed as delta diameter in comparison to baseline (no-flow condition, 0 mmHg). The endothelium-independent response was tested using the NO donor sodium nitroprusside (SNP; 10^−6^ mol/L, Sigma-Aldrich, Taufkirchen, Germany) and endothelium-dependent vasodilation was tested using acetylcholine (ACh; 10^−6^ mol/L, Fluka). Vascular responses to ACh and SNP were assessed in pressurized carotid arteries (no-flow condition). At the end of the protocol, vessels were perfused with Ca^2+^-free PSS solution (maximal diameter).

### 4.3. In Situ Evaluation of Nitric Oxide (NO) Levels in Carotid Arteries by Fluorescence Microscopy

Evaluation of the levels of NO in isolated carotid arteries was conducted as a separate group of experiments according to the established protocol described by Mihaljevic et al., and Matic et al. [5,57]. Carotid arteries were isolated and cannulated on pressure myograph with flow at 100 mmHg or without flow in the absence or presence of the nitric oxide synthase (NOS) inhibitor L-NAME, with the purpose of recording direct fluorescence measurements of endothelial NO production. Following a 30 min incubation period, carotid arteries were placed in HEPES buffer (137 mM NaCl; 5.4 mM KCl; 4.2 mM NaHCO_3_; 3 mM Na_2_HPO_4_; 0.4 mM KH_2_PO_4_; 0.5 mM MgCl_2_ × 6H_2_O; 0.8 mM MgSO_4_ × 7H_2_O; 10 mM glucose; 20 mM HEPES; and 1.2 mM CaCl_2_xH_2_O, pH 7.4) and loaded with 5 μM DAF-2DA (4,5-diaminofluorescein, Cayman Chemical, Michigan, USA) and incubated for 45 min at 37 °C, Zeiss Axioskop MOT2 microscope with an Olympus DP70 camera (excited at 490 nm and collected through a 530 nm band-pass emission filter) was used to record NO green fluorescence. The images were processed and analyzed by Image J software [58,59].

### 4.4. Biochemical Serum Analysis

In order to analyze serum levels of several biochemical components, blood samples were collected in tubes free of anticoagulants and kept at room temperature for at least one hour prior to centrifugation at 1000× *g* for 10 min at +4 °C. Following centrifugation, supernatants were collected and stored at −80 °C until analysis. Serum levels of total cholesterol, high-density lipoprotein (HDL), low-density lipoprotein (LDL), triglycerides (TG), glucose, aspartate transaminase (AST), alanine transaminase (ALT), urea, total proteins (TP), uric acid, alkaline phosphatase, and C-reactive protein (CRP) were determined in a routine laboratory using Architect c8000 instrument (Abbot).

### 4.5. Proteomic Analysis of Carotid Artery Tissue

#### Protein Extraction and Sample Preparation

Protein extraction from carotid arteries was performed using Minute Kit according to manufacturer’s instructions. Concentrations of total proteins in lysates were determined using a BCA protein Assay (Thermo Scientific, Rockford, IL, USA). Additionally, a pool of all samples was prepared by mixing equal protein amounts and used as internal standard (reference sample). Tandem mass tag TMT labelling was performed as described previously [60] In short, 20 µg of proteins from each sample were diluted using 0.1 M triethyl ammonium bicarbonate (TEAB, pH 7.8) up to a final volume of 50 µL. Samples were reduced (2.5 µL of 200 mM DTT, 1 h at 55 °C), alkylated (2.5 µL of 375 mM iodoacetamide IAA, 30 min at room temperature, in the dark) and precipitated using ice-cold acetone (−20 °C overnight). Following a centrifugation step (8000× *g*, 10 min at 4 °C), protein pellets were dissolved in 50 µL of 0.1 M TEAB containing trypsin (1:40, *w*/*w*) and digested overnight at 37 °C. Tryptic peptides were labelled using TMT tenplex reagents (Thermo Scientific, Rockford, IL, USA) which were prepared according to manufacturer’s instructions. For labelling, an amount of 19 µL was used and the reaction was quenched using 5% hydroxylamine. TMT-labelled peptides were combined into a new tube, aliquoted, dried and stored at −80 °C for LC-MS/MS analysis.

### 4.6. LC-MS/MS Analysis

High-resolution label-based LC-MS/MS analysis was performed in order to obtain protein identities and relative quantification data as reported previously [58]. In short, dried TMT-labelled peptides were dissolved in loading buffer (2% ACN in 0.1% FA) and loaded onto the trapping column by Ultimate 3000 RSLSnano system (Dionex). For the peptide separation, PepMap™ RSLC C18 (50 cm × 75 μm ID) column and 2 hours’ linear gradient of 5–35% buffer B (0.1% FA in 80% ACN) at a flow rate of 300 nL/min. Eluent was introduced into the Q Exactive plus mass spectrometer (Thermo Fisher Scientific, Rockford, IL, USA) by using nanospray Flex ion source and stainless steel emitter (New Objective). The ionization voltage was set to 2.1 kV and the ion transfer tube temperature was 250 °C. The analysis was performed in DDA positive ion mode using Top 8 method. Full scan FTMS spectra were acquired in mass range m/z 350.0 to m/z 1900.0 with the maximum injection time 110 ms, resolution 70,000 and AGC target 1 × 10^6^. For HCD fragmentation, step collision energy was set to 25, 35 and 40% NCE, resolution 17500 and AGC target 2 × 10^5^. An isolation window of ± 2.0 Da was applied to isolate precursor ions with the dynamic exclusion of 30 s. Precursor ions with the charges +1, as well as more than +7 were excluded from the fragmentation. The lock mass (m/z 445.1200) enabled internal calibration.

### 4.7. Proteomic Data Analysis

Protein identification was performed using SEQUEST algorithm implemented within Proteome Discoverer software (version 2.3., Thermo Fisher Scientific, Rockford, IL, USA) and database search against Mus musculus FASTA files (NCBI database, downloaded 7 December 2017, 46105 entries). Search parameters were set as follows: two trypsin missed cleavage sites, precursor and fragment mass tolerances of 10 ppm and 0.05 Da, respectively; carbamidomethyl (C) as fixed peptide modification, oxidation (M) and TMT sixplex (K, peptide N-terminus) as dynamic modifications. At least two unique peptides and cut-off of 5% FDR were set to obtain reliable protein identification. The FDR was calculated for the entire data set using Percolator algorithm within Proteome Discoverer workflow, based on the search results against a decoy database. Reporter-based relative quantification, statistical analysis, as well as gene ontology and pathway analyses were performed within Proteome Discoverer software. Internal standard was used as reference sample. Abundance normalization based on total peptide amount and ratio calculation of proteins were performed. Biological replicates study using a non-nested design without missing value imputation was applied. For statistical analysis, *p*-values were calculated using unpaired *t*-test with subsequent Benjamini–Hochberg correction using a maximum adjusted *p*-value set to 0.05.

### 4.8. Liver mRNA Expression Studies with Real Time PCR

Total RNA was isolated from liver tissue samples of *Tff3^−/−^* and WT controls on a LS and a HS diet using RNeasy Mini Kit (Qiagen. 1.5 µg of RNA was transcribed into cDNA with High-Capacity cDNA Reverse Transcription Kit (Applied Biosystems, Barcelona, Spain). Quantitative reverse transcription polymerase chain reaction (qRT-PCR) was performed using SYBR Green I (Invitrogen) detection chemistry and on StepOnePlus™ Real-Time PCR System (Applied Biosystems, Barcelona, Spain). A list of specific primers and optimized PCR conditions are given in Table 4. The cycling conditions comprised 3 min polymerase activation at 95 °C and 40 cycles including 95 °C for 1 min, annealing temperature specific for each primer pair (Table 4.) for 30 s, and elongation step at 72 °C for 30 s. Melting curve analysis was employed to confirm single product amplification. Gene expression was analyzed based on ΔΔCt method by REST© software (Quiagen) and normalized to stable housekeeping genes, β-actin, and β2-microglobulin. Changes were represented as log_2_ (fold change). Oligonucleotides used for Q-PCR analysis are listed in Table 4.

### 4.9. Liver Protein Expression Analysis of eIF2α Activation with Western Blot

Liver samples from WT and *Tff3-*depleted mice on a LS and a HS diet were homogenized using RIPA buffer (50 mM TRIS HCL, pH8, 150 mM NaCl, 1 mM EDTA, 1% NP40, 1% sodium deoxycholate, 0.1% SDS) supplemented with phosphatase and protease inhibitors (Roche), sonicated (3 × 30 sec), and centrifuged at 16,000× *g* for 20 min. Supernatant was collected and total protein concentration was measured by BCA protein assay kit (Pierce, Thermo Fischer). Proteins (25 µg per lane) were separated by denaturing sodium dodecyl sulfate polyacrylamide gel electrophoresis (SDS-PAGE) and transferred to nitrocellulose membrane and blocked with 5% BSA in TBS-T. Activation of ER stress relevant proteins was detected with polyclonal anti-eIF2α (#9722) and anti-phospho eIF2α (#9721) from Cell Signaling Technology at dilution 1:1000 in 5% BSA/TBST incubated over night at 4 °C. Secondary antibody was goat anti-rabbit IgG-HRP antibody (#170-6515; Biorad) and chemiluminescence signals was detected with the Alliance 4.7 Imaging System (UVITEC, Cambridge). Amido Black staining was used as loading and normalization control. Signal intensity was analyzed with Image J 1.48v [61].

### 4.10. Histomorphological Analysis of the Aorta

Aortas of *Tff3-*depleted and WT mice on a LS and a HS diet were fixed in 4% paraformaldehyde, paraffin-embedded, and cut into 6 µm sections using a sliding microtome (Leica SM 2000R, Leica, Wetzlar, Germany). Slides were deparaffinized and stained with two staining methods—orcein stain for visualization of elastic fibers and picrosirius red stain for visualizing collagen fibers. 1% orcein in 70% ethanol was used, slides were kept in the solution for 3 h, differentiation was done in acid alcohol (1% HCl in 70% ethanol) for 5 min and saturated picric acid in 96% ethanol was used for background staining. Picrosirius red stain was performed according to Puchtler. Slides were examined under Zeiss Axioskop 2 FS MOT microscope (Zeiss, Oberkochen, Germany) and digital photographs were taken with Olympus DP70 digital camera (Olympus, Tokyo, Japan) under a 10× objective.

Digital images were processed in FIJI software (FIJI is Just Image J), a distribution of Image J 2 open-source image processing software [62,63]. For both staining techniques, the color threshold tool was successfully used to make black-and-white masks containing elastin on orcein images and collagen on picrosirius red images. Furthermore, digital images were processed in Gimp software (GNU Image Manipulation Program, The GIMP team, GIMP 2.10.10, www.gimp.org) in order to create color-coded masks denoting different layers and structures of aortae to be analyzed (e.g., media and adventitia of aortae). Black-and-white and color-coded masks were opened and analyzed in FIJI. Share of elastin fibers and share of collagen fibers in aortic wall tissue was calculated from the elastin or collagen surface divided with aortal wall tissue surface and expressed as %. Thickness of media and adventitia was expressed as % of aortic wall thickness. Elastic lamellae were counted and lumen surface was measured as well.

### 4.11. Experimental Stroke Model—Transient Occlusion of Middle Cerebral Artery (toMCAO) Experiments

These experiments were performed on all four groups of mice, i.e., *Tff3^−/−^* and WT on a LS and a HS diet. For ischemic lesion induction, we used the adapted intraluminal filament model previously described by [64,65]. Briefly, preoperatively, the animals were administered i.p. 0.2 mL saline and s.c. 0.03 mg/kg bodyweight Buprenorphine (Buprenovet, Bayer, Germany) for analgesia. Anesthesia was induced by placing the animal in the induction chamber with 4% isoflurane (Isoflurane, Abbott, UK) in a 30/70 oxygen/air mixture until spontaneous movement of body and vibrissae stopped. The anesthetized animal maintained with 1.5–2% isoflurane in a 30/70 oxygen/air mixture was then placed in a lateral position on a heating pad in order to maintain the mouse body temperature at 37 °C. After aseptic preparation of the surgical site, a small incision was made in the right temporal muscle in the rostral part of the temporal area, dorsal to the retro-orbital sinus. subsequently placed in a supine position, and a calibrated 1 mm diameter Laser probe (Moor Instruments, Millwey, Devon, UK) was placed against the skull to monitor cerebral blood flow changes of the MCA on a laser Doppler perfusion monitor (Moor Instruments, Millwey, Devon, UK) following filament placement. After disinfecting the operating field, a midline incision in the neck was made and the mandibular glands, paratracheal strap, and sternomastoid muscles were retracted to expose the left common (CCA), internal (ICA), and external (ECA) carotid arteries. Through a small incision in the CCA and a silicon rubber-coated 6-0 monofilament (Doccol Corporation, Sharon, MA, USA) was advanced though the ICA into the circle of Willis, until it blocked the blood flow to the middle cerebral artery. After 60 min of occlusion, animals were re-anesthetized and reperfusion was initiated by filament removal. Animals with cerebral blood flow <20% after occlusion and immediately before pulling catheter out were considered as with successfully achieved occlusion. The CCA was permanently ligated. Following surgery animals were treated with s.c. buprenorphine in order to achieve postsurgical pain relief. Food was softened with water in order that damage in the neck muscles necessary for chewing would not result in cachexia. Animals were weighted before and 24 h after surgery.

### 4.12. Neurological Scoring

To compare stroke severity between groups, a modified neurological deficit score was performed before and 24 h after toMCAO using a five-point scale as previously described by [66]. Normal motor function was scored as 0, flexion of the contralateral torso and forelimb on lifting the animal by the tail as 1, circling to the contralateral side but normal posture at rest as 2, leaning to the contralateral side at rest as 3, and no spontaneous motor activity as 4. Neurological deficit was assessed by an investigator blinded to the experimental groups.

### 4.13. Statistical Analysis

For the FID experiments, delta diameter (μm) was determined by subtracting baseline diameter (0 mmHg) from the diameter at each level of flow. The differences among groups were assessed using the two-way ANOVA test (GraphPad Prism 5). To analyze fluorescence data, histology data, biochemical parameters, One-way ANOVA was used. Student’s *t* test was used to test the differences in normally distributed numerical variables between the two groups, while in the case of deviations from the normal distribution, the Mann–Whitney U test was used (SigmaPlot version 11.2, Systat Software, Inc., Chicago, IL, USA). Results are presented as means ± SD, or means ± SEM (as notes within figures) and the level of significance was determined at *p* < 0.05. For proteomic analysis reporter-based relative quantification, statistical analysis, as well as gene ontology and pathway analyses were performed within Proteome Discoverer software, as noted in Materials and Methods section.

## 5. Conclusions

In conclusion, present study confirmed that *Tff3* depletion seems to partially impair vascular function and worsen the outcomes of stroke, which is moderately affected by HS diet. Thus, *Tff3-*depleted mice could be added as potentially novel model for vascular functional studies.

## Figures and Tables

**Table 1:** Comparison of biochemical parameters among the study groups.

		Group				ANOVA
Parameter	Unit	WT LS	*Tff3^−/−^* LS	WT HS	*Tff3^−/−^* HS	*p* =
Uric acid	µmol/L	189.8 ± 74.2	217.6 ± 54.2	164.1 ± 19.4	201.6 ± 25.9	0.157
Blood glucose level	mmol/L	6.23 ± 3.64	5.96 ± 2.97	6.99 ± 1.85	6.43 ± 3.04	0.954
AST	U/L	353.6 ± 216.4	487.3 ± 90.74	389.5 ± 214.6	413.3 ± 230.3	0.630
ALT	U/L	42.6 ± 21.1	48.7 ± 4.93	39.8 ± 11.1	39.8 ± 14.9	0.835
CRP	mg/L	0.14 ± 0.02	0.15 ± 0.03	0.12 ± 0.01	0.12 ± 0.01	0.054
Urea	mmol/L	5.18 ± 0.73	3.63 ± 0.87	4.62 ± 0.73	5.10 ± 1.57	0.233
Total cholesterol	mmol/L	4.03 ± 0.52	3.97 ± 0.47	3.46 ± 0.69	3.65 ± 0.45	0.253
LDL	mmol/L	1.66 ± 0.40	1.57 ± 0.55	1.32 ± 0.44	1.33 ± 0.21	0.423
HDL	mmol/L	1.92 ± 0.23	2.05 ± 0.10	1.64 ± 0.35	1.83 ± 0.38	0.295
Triglycerides	mmol/L	0.98 ± 0.21	0.77 ± 0.07	1.11 ± 0.58	1.07 ± 0.22	0.261
Alkaline phosphatase	U/L	26.8 ± 2.68	25.3 ± 5.69	34.8 ± 12.9	27.7 ± 4.18	0.263
Total proteins	g/L	55.0 ± 2.83	54.0 ± 3.46	52.3 ± 3.44	50.2 ± 6.43	0.352

Data are presented as mean ± SD; differences were tested using One-way ANOVA and *p* values ≤0.05 were considered significant.

**Table 2:** Results of proteomic analysis of the differentially expressed proteins in carotid arteries of *Tff3^−/−^* mice on high salt diet (HS) compared to WT mice on HS diet.

Corresponding Gene Symbol	Abudance Ratio	*p* Value
Hspd1	9.625	1.08 × 10^−15^
Nefl	4.248	1.08 × 10^−15^
Ngf	3.339	1.08 × 10^−15^
Krt18	3.299	1.08 × 10^−15^
Tubb2b	3.293	1.08 × 10^−15^
Nid1	3.207	1.08 × 10^−15^
Mpz	2.962	1.08 × 10^−15^
Krt18	2.727	3.59 × 10^−13^
Mpz	2.688	8.81 × 10^−13^
Klk1b26	2.683	9.04 × 10^−13^
Mbp	2.432	2.27 × 10^−10^
Plp1	2.389	5.80 × 10^−10^
Nefh	2.270	7.15 × 10^−09^
Mbp	2.193	3.89 × 10^−08^
Klk1b1	2.187	4.35 × 10^−08^
Cnp	2.138	1.23 × 10^−07^
Nefh	1.982	3.64 × 10^−06^
Hsp90ab1	1.892	2.31 × 10^−05^
Prph	1.86	4.48 × 10^−05^
Mbp	1.849	5.42 × 10^−05^
Arpc4	1.848	5.42 × 10^−05^
Arpc4	1.848	5.42 × 10^−05^
Tnnt3	1.824	6.44 × 10^−05^
Myom2	1.756	2.58 × 10^−04^
Klk1b11	1.742	3.41 × 10^−04^
Klk1b22	1.741	3.48 × 10^−04^
Prph	1.734	4.01 × 10^−04^
Cd9	1.682	0.0011
Klk1b9	1.677	0.0012
Hist2h4	1.661	0.0016
H1f0	1.655	0.0017
Prph	1.606	0.044
Mybpc2	1.533	0.017
Myh3	1.501	0.029
Rps26	1.479	0.042
Rps26; Rps26-ps1	1.479	0.042
Gm6654	1.479	0.042
Nid1	1.478	0.042
Hba-a2	0.623	0.0035
Krt82	0.336	1.08 × 10^−15^
Krt35	0.221	1.08 × 10^−15^

Abundance ratio was calculated for *Tff3^−/−^* HS relatively to WT HS group, *p* ≤ 0.05 was considered significant.

**Table 3:** The structural characteristics of aorta in *Tff3^−/−^* and WT mice on LS and HS diet.

	Lumen Area [μm^2^]	Elastin [%]	Media [%]	ADV [%]	Wall Surface [μm^2^]	Collagen [%]	Wall to Lumen Ratio [%]
**WT LS**	714,834.57 ± 128,116.62	52.908 ± 4.44	70.669 ± 3,71	29.331 ± 3.71	379,241.52 ± 51,995.64	32.23 ± 4.32	54.00 ± 9.19
**WT HS**	772,048.33 ± 100,916.49	59.53 ± 4.69 *	76.83 ± 4.77 *	23.165 ± 4.77	374,726.27 ± 54,361.98	28.51 ± 4.59	48.8 ± 6.58
***Tff3^−/−^*** **LS**	912,555.53 ± 256,729.08	60.74 ± 6.65 *	76.49 ± 3.90*	23.50 ± 3.90 *	345,104.12 ± 39,500.88	30.01 ± 4.66	39.9 ± 10.03 *
***Tff3^−/−^*** **HS**	739,867.67 ± 182,518.08	57.06 ± 4.56	77.48 ± 1.79 *	22.51 ± 1.79 *	362,517.18 ± 39,568.26	27.24 ± 2.45 *	53.5 ± 22.37

* *p* < 0.05—compared to WT LS.

**Table 4:** Oligonucleotides used for Q-PCR analysis.

Gene Symbol	Accession No.	Primer sequence Forward (5′-3′) Reverse (5′-3′)	Optimized PCR Condition (Annealing Temp/MgCl_2_)
**ER stress markers**			
ATF4	NM_009716.3	CCACTCCAGAGCATTCCTTTAG CTCCTTTACACATGGAGGGATTAG	59 °C; 3,5 mM
BIP	NM_001163434.1	GAGACTGCTGAGGCGTATTT CAGCATCTTTGGTTGCTTGTC	58 °C; 3,5 mM
CHOP	NM_007837.4	TTGAGCCTAACACGTCGATTAT CACTTCCTTCTGGAACACTCTC	58 °C; 3 mM
EDEM	NM_138677.2	TGAAAGCATGTGAGGGTAGTG GAGAGAAGGGAAGACAGGATAGA	61 °C; 3,5 mM
GRP94	NM_011631.1	AAGAATGAAGGAAAAACAGGACAAAA CAAATGGAGAAGATTCCGCC	58 °C; 3 mM
sXBP1	NM_008934.4	GAGTCCGCAGCAGGTG GTGTCAGAGTCCATGGGA	56 °C; 3 mM
**Cytokines**			
CXCL1	NM_008176.3	GTGTCAACCACTGTGCTAGT CACACATGTCCTCACCCTAATAC	61 °C; 3,5 mM
IL1α	NM_010554.4	CCTTACACCTACCAGAGTGATTT CCTTACACCTACCAGAGTGATTT	65 °C; 3 mM
IL1β	NM_008361.4	ATGGGCAACCACTTACCTATTT GTTCTAGAGAGTGCTGCCTAATG	64 °C; 3 mM
IL6	NM_031168.2	GATAAGCTGGAGTCACAGAAGG TTGCCGAGTAGATCTCAAAGTG	59 °C; 3,5 mM
MCP1	NM_011333.3	CCTGGATCGGAACCAAATGA CGGGTCAACTTCACATTCAAAG	62 °C; 3 mM
TGFα	NM_031199.4	CTTTAGGAAGGACCTGGGTTG GTGTGTCCAGGCTCCAAATA	66 °C; 3 mM
TNFα	NM_013693.3	GTCTCAGAATGAGGCTGGATAAG CATTGCACCTCAGGGAAGAA	63 °C; 2,5 mM
**Oxidative Stress Markers**			
COX1	NM_008969.4	GTGCCAGAACCAGGGTGTCT GTAGCCCGTGCGAGTACAATC	58 °C 3 mM
GPX1	NM_008160.6	GGTTCGAGCCCAATTTTACA CATTCCGCAGGAAGGTAAAG	58 °C 2,5 mM
NOX2	NM_007807.5	ACTCCTTGGGTCAGCACTGG GTTCCTGTCCAGTTGTCTTCG	62 °C 3 mM
SOD1	NM_011434.2	GCCTTCTGCTCGAAGTGGAT GGAAGCATGGCGATGAAAGC	59 °C 3,5 mM
SOD3	NM_011435.3	TGGCTGATGGTTGTACCCTG TGAGAAGATAGGCGACACGC	60 °C 2,5 mM
**Housekeeping Genes**			
ActB	NM_007393.5	GCAAGCAGGAGTACGATGAG CCATGCCAATGTTGTCTCTT	61 °C; 3,5mM
B2M	NM_009735.3	CCTGCAGAGTTAAGCATGACAGT TCATGATGCTTGATCACATGTCT	60 °C; 3mM

ATF4, Activating transcription factor 4; BIP, Endoplasmic Reticulum Chaperone BiP; CHOP, DNA Damage-Inducible Transcript 3; GRP94, Heat Shock Protein 90 Beta Family Member 1; sXBP1, Spliced X-box Binding Protein 1; CXCL1, C-X-C Motif Chemokine Ligand 1; IL1α, Interleukin 1 Alpha; IL1β, Interleukin 1 Beta; IL6, Interleukin 6; MCP1, Monocyte Chemoattractant Protein-1; TGFα, Tumor Growth Factor Alpha; TNFα, Tumor Necrosis Factor Alpha; COX1, Cyclooxygenase 1; GPX1, Glutathione Peroxidase 1; NOX2, NADPH oxidase 2, SOD1, Superoxide Dismutase 1; SOD3, Superoxide Dismutase 3; ActB, Actin Beta; B2M, Beta-2-Microglobulin.

## Supplementary Materials

Supplementary materials can be found at https://www.mdpi.com/1422-0067/20/20/5188/s1.
