# Supplementary material for: Impact of High Salt Diet on Cerebral Vascular Function and Stroke in Tff3−/−/C57BL/6N Knockout and WT (C57BL/6N) Control Mice
Source: Int J Mol Sci. 2019 Oct 19;20(20):5188. doi: 10.3390/ijms20205188 (PMC6829871; doi:10.3390/ijms20205188)
Supplement: Supplementary file 1 [file ijms-20-05188-s001.pdf]

International Journal of
Molecular Sciences

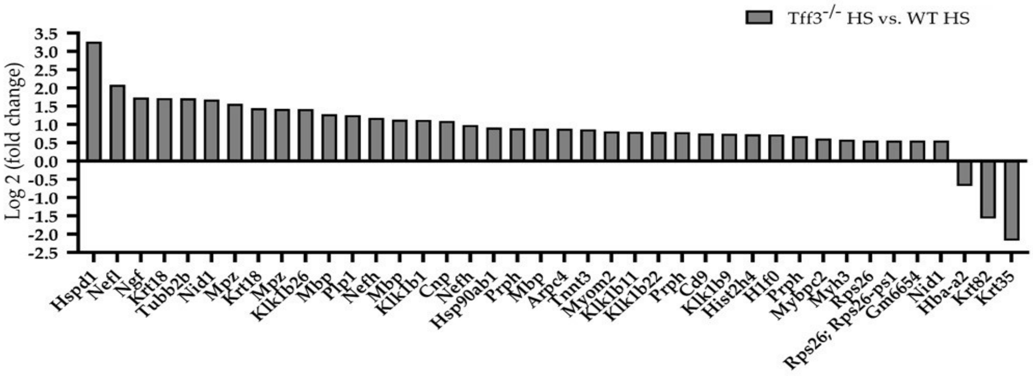

Supplementary Figure 1. Abundance ratio of proteins isolated from carotid arteries of *Tff3*$^{-/-}$ HS and WT HS mice. Data are expressed as log (2) of abundance ratio.
